# Effects of musical expertise on line section and line extension

**DOI:** 10.3389/fpsyg.2023.1190098

**Published:** 2024-04-09

**Authors:** Yilai Pei, Zhiyuan Xu, Yibo He, Xinxin Liu, Yuxuan Bai, Sze Chai Kwok, Xiaonuo Li, Zhaoxin Wang

**Affiliations:** ^1^Key Laboratory of Brain Functional Genomics, Ministry of Education and Shanghai, Institute of Cognitive Neuroscience, School of Psychology and Cognitive Science, East China Normal University, Shanghai, China; ^2^Shanghai Changning Mental Health Center, Shanghai, China; ^3^Shanghai Key Laboratory of Magnetic Resonance, East China Normal University, Shanghai, China; ^4^Phylo-Cognition Laboratory, Division of Natural and Applied Sciences, Data Science Research Center, Duke Institute for Brain Sciences, Duke Kunshan University, Kunshan, Jiangsu, China; ^5^Institute of Research of Musical Arts, Shanghai Conservatory of Music, Shanghai, China

**Keywords:** line bisection, pseudoneglect, line extension, spatial evaluation, musicians

## Abstract

**Background:**

This study investigated whether music training led to better length estimation and/or rightward bias by comparing the performance of musicians (pianists) and non-musicians on performance of line sections and line extensions.

**Methods:**

One hundred and sixteen participants, among them 62 musicians and 54 non-musicians, participated in the present study, completed line section and line extension task under three conditions: 1/2, 1/3 and 2/3.

**Results:**

The mixed repeated measures ANOVA analysis revealed a significant group × condition interaction, that the musicians were more accurate than non-musicians in all the line section tasks and showed no obvious pseudoneglect, while their overall performance on the line extension tasks was comparable to the non-musicians, and only performed more accurately in the 1/2 line extension condition.

**Conclusion:**

These findings indicated that there was a dissociation between the effects of music training on line section and line extension. This dissociation does not support the view that music training has a general beneficial effect on line estimation, and provides insight into a potentially important limit on the effects of music training on spatial cognition.

## Introduction

Spatial skills are essential for everyday life. Manual line bisection is a commonly used method to assess visuospatial neglect in neurological patients and is expected to be related to spatial estimation abilities. Interestingly, when individuals attempt to identify the midpoint of horizontal lines, they often misbisect, typically erring to the left of the true center [for an in-depth review, see (Jewell and McCourt, 2000)]. This phenomenon has been termed “pseudoneglect” (Bowers and Heilman, 1980). The prevailing explanation for these leftward biases is the notion of right hemispheric dominance for spatial attention (Fink et al., 2000). According to this theory, spatial information is predominantly processed in the right hemisphere, as supported by imaging studies (Witelson, 1976; Ratcliff, 1979; Cicek et al., 2003, 2007, 2009). Consequently, there is an attentional preference and overestimation for stimuli presented in the left hemispace since they are processed in the right hemisphere due to the optic chiasm. This explanation is also consistent with clinical findings. For instance, lesions in the right hemisphere are associated with visual–spatial agnosia (Mc et al., 1950; Lawson, 1962). One implication of this is that expertise that can influence brain plasticity related to spatial cognition may lead to changes in spatial estimation.

Previous studies have shown that exposure to music, including listening to music (even white noise), and music training can enhance spatial cognition. For instance, studies have demonstrated that the simultaneous binaural presentation of auditory white noise can affect participants’ performance in visual and haptic bisection tasks, resulting in a reduction of leftward errors (Cattaneo et al., 2012). Furthermore, research using tasks like the Paper Folding and Cutting task has indicated that preschool children who received 8 months of music lessons (even just listening to K448 for 8 min) performed better in spatial reasoning compared to demographically similar groups (Rauscher et al., 1993, 1994). Additional studies have consistently shown that musicians, in general, exhibit superior spatial cognition when compared to non-musicians. This advantage extends to various domains, including visuospatial span (Amer et al., 2013), Benton Judgment of Line Orientation (Strong and Mast, 2019), visual–spatial sequence learning (Anaya et al., 2017), spatial memory (Suarez et al., 2016), visual threshold (Weiss et al., 2014), and more, for a meta-analysis, (see Voyer and Jansen, 2017). Moreover, imaging studies have revealed differences in gray matter volume and microstructural white matter in right visual–spatial brain regions, such as the right frontal gyrus, when comparing professional musicians, both young and old, with matched amateur musicians or non-musicians (Gaser and Schlaug, 2003). These studies collectively suggest that line estimation is not purely a perceptual task but is subject to the influence of cognitive processes. Music training may indeed contribute to improved spatial estimation abilities more broadly.

To our knowledge, only three studies have delved into the effects of music expertise on spatial estimation (Patston et al., 2006; Agrillo and Piffer, 2012; Lega et al., 2014). Interestingly, two of these studies focused on the bisection task (Patston et al., 2006; Lega et al., 2014), but their results were inconclusive. In one study by Patston et al. (2006) musicians exhibited a slight rightward bias, while non-musicians displayed a leftward bias. Furthermore, musicians demonstrated more accurate line bisecting abilities compared to non-musicians (Patston et al., 2006). Agrillo and Piffer (2012) also found that musicians outperformed non-musicians in estimating the magnitude of space, where participants had to determine which of two presented lines was longer. However, Lega et al. (2014) reported a significant rightward bias for both musicians and non-musicians in a bisection task when listening to white noise, and the overall standard deviations were similar between the two groups. Clearly, additional research is warranted to address these limited yet conflicting findings, including further investigation into whether musicians do indeed exhibit a rightward bias.

Another crucial point to consider is that previous studies on length estimation in musicians have typically focused on measurements within a specific range, such as line bisection or discriminating which line is longer between two lines. Nevertheless, it’s important to recognize that line extension is a fundamental aspect of length estimation. Surprisingly, to the best of our knowledge, only seven studies have explored line extension in both healthy participants and patients (Ishiai et al., 1994a,b; Chokron et al., 1997; Bisiach et al., 1998; Perri et al., 2000; Geminiani et al., 2002; Charras et al., 2010). Typically, these studies involved providing participants with a half-line and instructing them to complete it to form a whole line with two equal halves (Ishiai et al., 1994a; Chokron et al., 1997; Geminiani et al., 2002). Interestingly, it was observed that patients with left unilateral neglect tended to overextend the line, drawing it longer than necessary. Strikingly, this overextension did not significantly differ from the performance of control participants (Ishiai et al., 1994a,b), and in some instances, it was even more pronounced (Bisiach et al., 1998). These findings, however, appeared to contradict the explanations for results in line bisection tasks, where patients were expected to underextend the line (Savazzi et al., 2007). Savazzi has proposed the intriguing idea that there may be anisometry in the representation of space and lines, particularly in the context of neglect dyslexia (Savazzi et al., 2004), and this phenomenon might extend to healthy individuals as well (Ishiai et al., 1994a,b). If this is indeed the case, it raises the possibility that the effects of musical expertise on line extension may differ from those on line bisection or trisection tasks.

One more consideration regarding the bisection task is that performance can be influenced by various confounding factors, such as inter-individual variability and language (Jewell and McCourt, 2000; Learmonth and Papadatou-Pastou, 2022; Kaul et al., 2023). For instance, researchers have noted significant inter-individual variability in the direction and extent of bisection errors, with approximately half of the subjects deviating to the right and the other half to the left of the true midpoint (Halligan et al., 1990; Manning et al., 1990). This inter-individual variability can complicate the interpretation of results and should be carefully controlled. In the present study, two approaches were employed to mitigate this potential complication by “predefining” the spatial bias. The first approach involved displaying stimuli in the left hemispace. For example, in seven tactile and visuotactile studies, Bradshaw et al. found greater leftward errors when stimuli were presented in the left hemispace (Bradshaw et al., 1983). The second approach was the use of cues. Most authors reported that bisections deviated from the veridical midpoint in the direction of the cued end, compared to control conditions with no cues (Milner et al., 1992). To address potential language-related effects, native Chinese speakers were selected as participants in this study.

The aims of the present study were twofold: (1) to investigate the effects of music expertise on line section and line extension tasks; and (2) to explore whether there was anisometry in line representation between line section and line extension tasks in both musicians and non-musicians. Participants were tasked with dividing or extending line segments by 1/3, 1/2, or 2/3, a task akin to number line estimation, which has been demonstrated to strongly correlate with math problem-solving (Zhu et al., 2017).

## Methods

### Participants

One hundred and sixteen participants were recruited for the present study. For the assigned sex at birth between male and female, one-hundred and six reported as females and nine as males. Among them, there were 62 musicians (from the Shanghai Conservatory of Music, 50 females, aged 22.6 ± 3.4, range = 18–35) with an average of 13.0 ± 4.5 years of piano training (range = 5–25 years) and 54 non-musicians (all from East China Normal University, 46 females, aged 23.0 ± 1.8, range = 19–28) without professional music training. Thirty-six musicians majored in piano, while others had a minor in piano with a minimum of 5 years of piano training. Among them, 24 were from the Department of Vocal Studies, 1 from the Department of Conducting, and 1 majored in Music Psychology. Only one nonmusician had 0.5 years of piano training. There were no significant group differences in age (*t* = 0.105, *p* = 0.92) or sex (*χ*^2^ = 0.552, *p* = 0.46). All participants were native Chinese speakers and right-handed, except for one non-musician who was ambidextrous, and 3 musicians who were left-handed, with 1 musician being ambidextrous. Normal hearing and normal or corrected-to-normal vision were reported. Participants were compensated for their time. Written informed consent was obtained from all participants, and the present study was approved by the local ethics committee of East China Normal University.

Note that we originally conducted the study with *N* = 40 (21 musicians). Following reviewer comments and a request for a power analysis, a power analysis was conducted using G*Power version 3.1.9.7 [44] to determine the minimum sample size required to test the study hypothesis. The results indicated that the required sample size to achieve 80% power for detecting a medium effect size (0.25) at a significance criterion of *α* = 0.05 was *N* = 86 for repeated measures with 3 factors in 2 groups. Accordingly, we collected additional participants. Note the power analysis is not a retrospective analysis (Hoenig and Heisey, 2001; Kovacs et al., 2022), as it was carried out before the collection of new additional participants.

### Procedure

Participants were asked to complete a series of length section/extension tasks on paper. In these tasks, participants were instructed to divide or extend line segments by 1/3, 1/2, or 2/3. To be specific, in the section tasks, the start and end points of the lines were described using three types of symbols: fractions, integers, and music scales. These symbols were presented in a pseudorandom order to minimize the effects of habituation and to prevent the development of a consistent strategy.

Each type of symbol was displayed on a separate page. The start and end points of the line were marked with integers or music scales, and a corresponding fraction/number/symbol cue for 1/3, 1/2, and 2/3 was provided before the line. Participants were then asked to mark the location on the line based on the given cue using a pencil. For instance, the start and end points could be represented as ‘1’ or ‘Do’ and ‘7’ or ‘Ti,’ and the section cues could be ‘3’ or ‘Mi,’ ‘4’ or ‘Fa,’ and ‘5’ or ‘So,’ corresponding to 1/3, 1/2, and 2/3 of the line, respectively. In the fraction condition, there were no start or end symbols.

**Figure 1:**
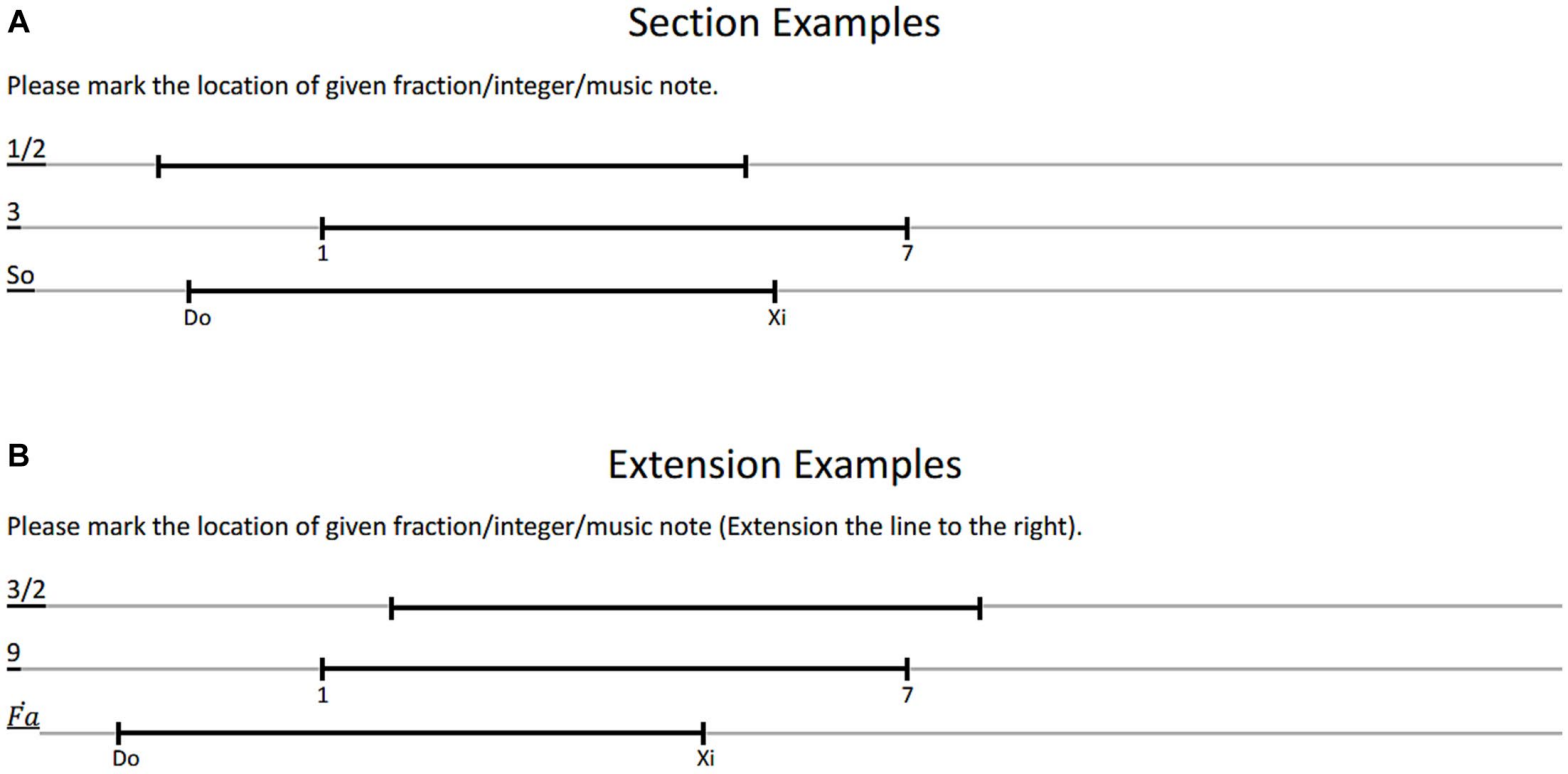
Examples of line section task and line extension tasks. All stimuli were presented pseudorandomly at the left side of a paper, with three kinds of cues (i.e., fractions, integers and music scales) and corresponding markers under the line segment. In the section task, participants were asked to divide the line segment by 1/3, 1/2, or 2/3. In the extension task, participants were asked to extend the line segments by 1/3, 1/2, or 2/3.

Each page contained four trials presented in pseudorandom positions on the left side of the page, with line lengths of approximately 10 cm. Overall, the materials for these tasks consisted of 4 A4 pages in a session, each containing 12 horizontal lines, with four trials for each condition (as shown in Figure 1A).

Next, participants were asked to perform an extension task. The stimuli and symbol types were the same as in the section task, except that the cues were changed to 3/2, 4/3, and 5/3 for the fraction condition, 9, 10, and 11 for the integer condition, and high Mi, high Re, and high Fa for the musical note condition, respectively (see Figure 1B). Participants were instructed to mark the corresponding point on the right side of the line using a pencil. For example, in the musical note condition for a 3/2 extension, a line segment with the start point ‘Do’ and end point ‘Xi’ was provided, and participants needed to mark the possible location of ‘high Fȧ.’ Similar to the section tasks, the materials for these extension tasks also consisted of 4 A4 pages, each containing 12 horizontal lines displayed in pseudorandom positions. Two additional section/extension sessions followed, resulting in a total of two section sessions and two extension sessions. Participants completed all tasks in approximately half an hour.

### Data analysis

A mixed repeated measurement 2 (Between-subject factor: musicians vs. non-musicians) × 2 (Within-subject factor, Condition: line section vs. line extension) × 3 (Within-subject factor, 3 levels) ANOVA was used to analyze the normalized deviations between the marked location and the veridical location using SPSS Statistics (Ver 23). Line section and extension errors were measured with approximation to the nearest millimeter and the normalized deviation was used, which was calculated with the formula of $\mathrm{Deviation}=100\%\times \left({\mathrm{Point}}_{\mathrm{marked}}-{\mathrm{Point}}_{\mathrm{veridical}}\right)/{\mathrm{Length}}_{\mathrm{total}}$ . For *post hoc* analysis, a further repeated measures 2 (Between-subject factor: musicians vs. non-musicians) × 3 (Within-subject factor 2, Levels) ANOVA and *T*-test (two-tailed) was used for line section condition and line extension condition separately. A further correlation analysis was also applied to investigate the possible relationship between section tasks and extension tasks. The threshold was set at *p* = 0.05. Based on PauTa Criterion, data exceeding three sigma of the mean (non-recursive) was excluded. Bonferroni correction was used to control type I error due to multiple comparison.

## Results

### Results of the mixed repeated measures ANOVA

**Figure 2:**
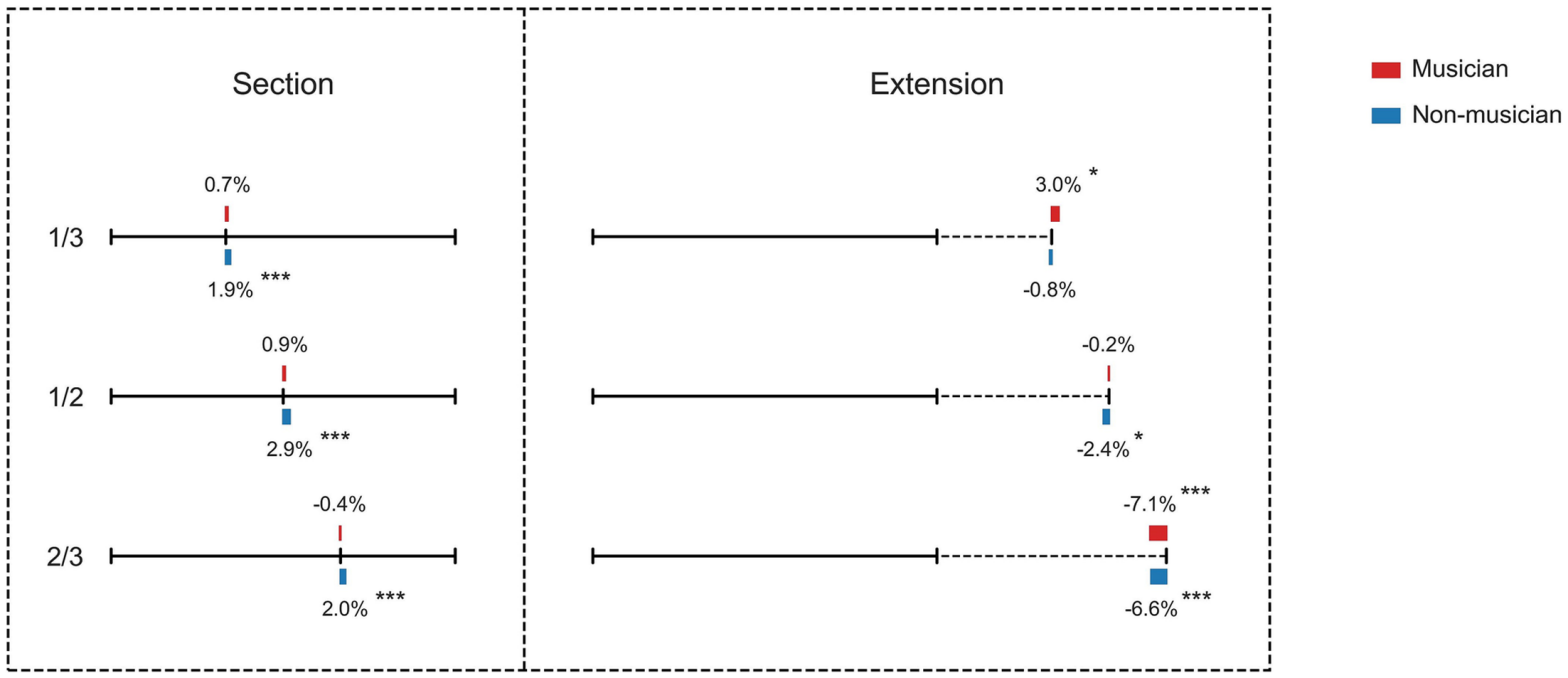
Behavioral results of section tasks (left) and extension tasks (right). The mean normalized deviations (vs. veridical point) of the musicians (red) and the non-musicians (blue) were shown. *p < 0.05; **p < 0.01; ***p < 0.001, Bonferroni corrected.

The detailed performance was listed in Table 1; Figure 2. The mixed repeated-measures ANOVA revealed that there were significant main effects of conditions (*F*(1,228) = 36.0, *p* < 0.001, *η*^2^ = 0.240), levels (*F*(2,228) = 100.0, *p* < 0.001, *η*^2^ = 0.467), but not groups (*F*(1,114) = 0.002, *p* = 0.967, *η*^2^ = 0.001). Moreover, there was a significant condition by group interaction (*F*(1,228) = 9.05, *p* = 0.003, *η*^2^ = 0.074), and a level by group interaction (*F*(2,228) = 9.92, *p* < 0.001, *η*^2^ = 0.080), and a condition by level interaction (*F*(2,228) = 77.7, *p* < 0.001, *η*^2^ = 0.405). There was also a three way condition by level by group interaction (*F*(1,228) = 3.64, *p* = 0.028, *η*^2^ = 0.031).

**Table 1:** The averaged normalized deviations (%).

	Musicians		Non-musicians		Group differences	
	Mean(SD)	*p*	Mean(SD)	*p*	t	*p*
Section						
1/3	0.52(2.9)	0.32	1.9(3.1)	<0.001	−2.6	0.024
1/2	0.88(3.6)	0.16	2.7(3.5)	<0.001	−2.7	0.016
2/3	−0.13(2.7)	1	2.0(3.0)	<0.001	−4.1	0.001
Extension						
1/3	2.6(6.3)	0.004	−1.2(4.9)	0.13	3.6	0.001
1/2	−0.63(5.6)	0.76	−2.9(4.3)	<0.001	2.4	0.038
2/3	−7.1(8.7)	0.002	−7.1(7.0)	<0.001	−0.023	1

Data format: mean(SD); Bonferroni corrected.

### Results of the line section conditions

For the section conditions, the *post hoc* repeated-measures ANOVA with group (musicians and non-musicians) as between-subject factor revealed a significant main effect of group (*F*(1,114) = 14.4, *p* < 0.001, *η*^2^ = 0.112). Further *post hoc t*-tests revealed that the non-musicians displayed significant leftward bias in all line section conditions, while no significant errors were found in musicians in all line section conditions. Moreover, the differences between musicians and non-musicians were significant in all the three conditions. The statistical results were listed in Table 1. A main effect of level was also found (*F*(2,228) = 5.09, *p* = 0.007, *η*^2^ = 0.043), with participants showed significant bigger deviation in the bisection condition vs. 2/3 trisection condition (*t*_116_ = 3.4, *p* = 0.001), in line with the Weber-Fechner law that the noticeable difference depended on the magnitude or intensity of the stimulus. No significant group by level interaction was found (*F*(2,228) = 1.78, *p* = 0.17, *η*^2^ = 0.015). The deviations were also shown in Figure 2.

### Results of the line extension conditions

For the extension conditions, a significant main effect of level was found (*F*(2,228) = 125, *p* < 0.001, *η*^2^ = 0.524). A further *post hoc* t-test revealed that the normalized deviations were significant in all the three line extension conditions. A further analysis revealed that the leftward errors increased in the 1/2 condition (vs. 1/3, *t*_112_ = 6.9, *p* < 0.001) and 2/3 condition (1/3 vs 2/3, *t*_112_ = 12.0, *p* < 0.001; 1/2 vs 2/3, *t*_112_ = 12.4, *p* < 0.001). No significant main effect of group (*F*(1,114) = 2.45, *p* = 0.12, *η*^2^ = 0.021) was found. Yet, a significant group by condition interaction (*F*(2,228) = 9.12, *p* < 0.001, *η*^2^ = 0.074) was found. A further *post hoc t*-test revealed non-musicians performed better in the 1/3 condition than musicians did, while musicians performed better in the 1/2 extension condition. In the 2/3 extension condition, both groups displayed leftward errors and no significant group difference was found. The statistical results were listed in Table 1.

### Correlations

**Figure 3:**
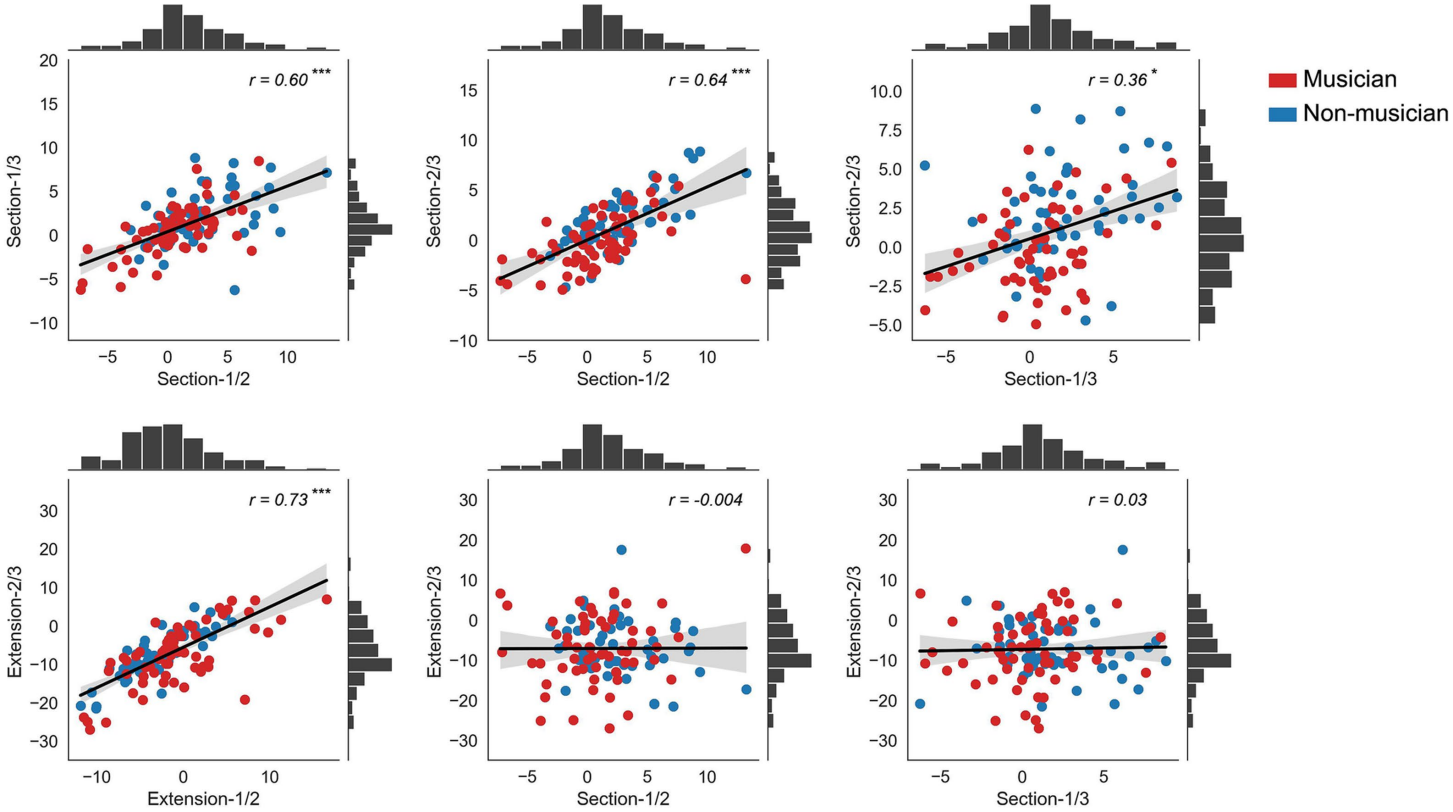
Results of correlation analysis of all participants. Significant correlations were found between the performance within section conditions, and between the performance within extend conditions, but not between the performance on extension conditions and section conditions. The histograms of frequency were also illustrated. *p < 0.05; **p < 0.01; ***p < 0.001; Bonferroni corrected.

The correlation matrix was presented in Table 2 (Musicians), Table 3 (Non-musicians), and Table 4 (All Participants). Among the section conditions, significant correlations were found between the mean normalized deviations of the bisection condition and the other two trisection conditions for both groups. Regarding the extension conditions, significant correlations were identified through pairwise correlation analyses in both groups. However, between the section conditions and extension conditions, no significant correlations were observed for both groups. The combined results from both musicians and non-musicians were also computed, revealing a similar pattern as shown in Table 3 and illustrated in Figure 3. To mitigate the risk of type I errors associated with multiple comparisons, Bonferroni correction was applied.

**Table 2:** Correlation (Pearson’s *r*) matrix of musicians.

	Section			Extension		
	1/3	1/2	2/3	1/3	1/2	2/3
Section						
1/3	/	0.67(<0.001)	0.36(0.025)	0.08(1)	0.03(1)	0.09(1)
1/2	0.67(<0.001)		0.50(<0.001)	0.22(0.45)	−0.07(1)	0.17(0.89)
2/3	0.36(0.025)	0.50(<0.001)		−0.05(1)	−0.004(1)	−0.03(1)
Extension						
1/3					0.78(<0.001)	0.48(<0.001)
1/2				0.78(<0.001)		0.71(<0.001)
2/3				0.48(<0.001)	0.71(<0.001)	

Data format: *r*(p). Bonferroni corrected.

**Table 3:** Correlation (Pearson’s *r*) matrix of non-musicians.

	Section			Extension		
	1/3	1/2	2/3	1/3	1/2	2/3
Section						
1/3	/	0.47(<0.001)	0.26(0.28)	0.12(1)	−0.08(1)	−0.06(1)
1/2	0.47(<0.001)	/	0.74(<0.001)	0.008(1)	−0.30(0.15)	−0.24(0.25)
2/3	0.26(0.28)	0.74(<0.001)	/	−0.22(0.5)	−0.34(0.07)	−0.16(1)
Extension						
1/3				/	0.66(<0.001)	0.45(0.004)
1/2				0.66(<0.001)	/	0.86(<0.001)
2/3				0.45(0.004)	0.86(<0.001)	/

Data format: Pearson’s *r*(p). Bonferroni corrected.

**Table 4:** Correlation (Pearson’s *r*) matrix of all participants.

	Section			Extension		
	1/3	1/2	2/3	1/3	1/2	2/3
Section						
1/3	/	0.60(<0.001)	0.36(0.025)	0.02(1)	−0.06(1)	0.03(1)
1/2	0.60(<0.001)		0.64(<0.001)	0.04(1)	−0.22(0.1)	0.004(1)
2/3	0.36(0.025)	0.64(<0.001)		−0.22(0.1)	−0.20(0.15)	−0.08(1)
Extension						
1/3					0.75(<0.001)	0.44(<0.001)
1/2				0.75(<0.001)		0.73(<0.001)
2/3				0.44(<0.001)	0.73(<0.001)	

Data format: Pearson’s *r*(p). Bonferroni corrected.

## Discussion

In the present study, we aimed to test the hypothesis that musicians exhibit superior line estimation skills in both line section tasks and line extension tasks. A significant group by condition interaction was observed. Specifically, we found that musicians demonstrated more precise line division compared to non-musicians, although no rightward bias was detected. However, there was no significant difference between the two groups in the extension tasks, with musicians only displaying higher accuracy in the 1/2 condition compared to non-musicians. Furthermore, our correlation analyses revealed significant correlations between the bi/trisection conditions and between the extension conditions. However, no significant correlations were identified between the section conditions and extension conditions.

### Performance of line section conditions

Our findings in the bisection condition align with Patston et al.’s (2006) discovery that musicians exhibited more accurate line bisecting abilities compared to non-musicians. Furthermore, our study provides additional evidence that musicians demonstrated greater accuracy when trisecting lines than their nonmusician counterparts. However, it’s noteworthy that we did not detect a significant rightward bias in either musicians or non-musicians in the bi/trisection conditions. Instead, non-musicians displayed a consistent leftward bias in all three conditions. These results suggest that the two approaches used to “predefine” spatial bias were effective in non-musicians (Halligan et al., 1990; Manning et al., 1990; Chieffi et al., 2012), thereby reducing the potential influence of inter-individual variability on line estimation. Nevertheless, among musicians, we did not observe significant differences between the marked locations and the veridical locations. These findings indicate that musicians, at least those included in this study, possessed superior spatial representations of line segments compared to non-musicians, and that musicians performed better when segmenting lines than non-musicians did.

### Performance of line extension conditions

In the extension conditions, non-musicians exhibited leftward errors in the 1/2 and 2/3 conditions. It’s worth noting that these results were inconsistent with findings from previous studies in which participants, including patients with left unilateral neglect, tended to overextend the line when tasked with completing the half-line to create a whole line with two equal halves (Ishiai et al., 1994a,b; Bisiach et al., 1998). We speculate that the “enhancement” theory (Ishiai et al., 1994a,b; Bisiach et al., 1998) can be applied to explain these discrepancies. According to this theory, when participants are engaged in the task of completing the half-line to form a whole line, it essentially becomes a straightforward copying task. As the perceptual salience of the given stimulus or line is enhanced, participants tend to overestimate the given line, leading to an overextension of the copied line.

Interestingly, although no significant differences in performance on the extension tasks were found between musicians and non-musicians, a repeated measures ANOVA unveiled a group by level interaction. Subsequent *post hoc* analysis showed that musicians exhibited a rightward error in the 1/3 extension condition and a leftward error in the 2/3 condition. However, no significant bias was observed in the 1/2 extension condition. These results indicate that musicians hold an advantage specifically in the 1/2 extension condition, but they do not support the notion that music training universally enhances line estimation skills.

### Possible mechanisms

To explore possible underlying mechanisms, we conducted a correlation analysis between performance on the bi/trisection tasks and extension tasks. Our rationale was that if the same mechanism underlies both tasks, adherence to the Weber-Fechner law should be evident. This would mean that deviations should be correlated between different conditions because the noticeable difference depends on the magnitude or intensity of the stimulus.

The correlation analyses revealed distinct patterns between the performance on section tasks and extension tasks. Notably, deviations were significantly correlated within the section conditions for both musicians and non-musicians, as well as within the extension conditions. These results suggest that our data were consistent and that a similar mechanism likely operated among the three section conditions and among the three extension conditions. However, we did not observe any significant correlations between performance on the section tasks and extension tasks in both musicians and non-musicians after applying Bonferroni Correction. These findings support Savazzi et al.’s (2004) argument that there is anisometry in space/line representation between line bisection and line extension.

We speculate that the differences in performance between non-musicians and musicians can be attributed to their long-term music training. This idea aligns with the findings of Hausknecht et al. (2007), who argued that improvements in spatial tasks are more significant when the training demands similar abilities (Hausknecht et al., 2007). Voyer and Jansen (2017) also argued that motor expertise plays a potential role in promoting good spatial performance. In the case of music, there are clear associations between pitch tones and spatial concepts (Rusconi et al., 2005; Lidji et al., 2007; Klapman et al., 2021). Humans tend to represent pitch in a spatial format, and a classic example of this is the Spatial-Musical Association of Response Codes (SMARC) effect (Rusconi et al., 2005; Guilbert, 2020). This effect reflects faster responses to low-pitched tones when pressing a left/bottom-side key and to high-pitched tones when pressing a right/top-side key. Even non-musicians exhibit a subtle association between pitch height and horizontal space (Hartmann, 2017), albeit with a different explanation (Guilbert, 2020).

Di Stefano argued that both the metaphorical and literal uses of spatial concepts in describing music are rooted in the way people perceive the dynamic changes of acoustic features in terms of spatial phenomenology (Di Stefano, 2022). People consistently associate high-pitched sounds with objects located high up in space (Spence, 2011). Moreover, musical information can automatically orient attention according to specific spatial musical forms, as reported by Akiva-Kabiri et al. (2014) in musical space synesthetes. Importantly, these associations go beyond the auditory modality, extending to pitch and space relationships without sound (Ariga and Saito, 2019; Jiang and Ariga, 2020), as well as music notation and space (Fumarola et al., 2020). The perception of melodic ‘ups and downs’ even elicits spatial predictions that modulate the spatial processing of visual stimuli (Romero-Rivas et al., 2018). Imaging studies have revealed asymmetries in brain activities when reading clefs without auditory input, further supporting the link between pitch and space (D'Anselmo et al., 2018). Additionally, spatial ability is reported to be related to pitch processing. For example, there is a positive correlation between children’s spatial and pitch-matching skills, suggesting spatial underpinnings for pitch (Mohring et al., 2016). Amusia patients with difficulty in discriminating pitch changes are associated with deficits in spatial processing (Douglas and Bilkey, 2007), although some studies present differing results (Tillmann et al., 2010). Furthermore, superior pitch identification ability is linked to better mental rotation performance in non-musicians (Hou et al., 2023). Hence, the associations between music and space are bidirectional (Lachmair et al., 2019). Given musicians’ superior performance in pitch processing (Forgeard et al., 2008), it’s plausible that their better line estimation abilities in specific conditions are a result of their musical expertise. Therefore, their performance in the section tasks may be more accurate than that of non-musicians.

It appears that the complex results of the extension tasks cannot be attributed to a simple explanation. In these tasks, participants were required to draw a point beyond a spatial range. It seems that musicians may still possess a superior spatial representation due to their musical expertise, but this advantage may be limited to the 1/2 extension task. We further speculate that the “enhancement” theory (Ishiai et al., 1994a,b; Bisiach et al., 1998), which suggests overestimation of lines in the left hemispace, may still apply to both musicians and non-musicians. Consequently, the overall performance in the extension tasks, especially in the 2/3 extension condition, was comparable between the two groups of participants.

### Limitations

The first limitation is that the majority of our participants were females. Although we did not find significant a gender difference with a further repeated measures ANOVA, *F*(1,114) = 1.46, *p* = 0.229, *η*^2^ = 0.013, nor a significant condition by gender interaction, *F*(1,114) = 0.194, *p* = 0.660, *η*^2^ = 0.002, there might be gender difference that deserves further study, as males outperformed females in spatial ability (Yuan et al., 2019) but bisections were more leftward in studies with a higher percentage of boys relative to girls (Roig and Cicero, 1994; Kaul et al., 2023), despite no gender difference were reported in some other studies (Jewell and McCourt, 2000).

A second limitation is that there are advantages in working memory (Schulze et al., 2011) and visual working memory (Oechslin et al., 2013), symbol coding and perception (Chalas et al., 2022), general math cognition (Schmithorst and Holland, 2004), and audiovisual magnitude comparisons (Paraskevopoulos et al., 2014) in musicians compared to non-musicians. These factors may also play roles to explain our results, especially in the section conditions, yet the causal relationships between them deserve further investigation. We also note that other non-spatial factors, such as differences in intelligence, motivation, and/or manual dexterity, cannot be excluded.

## Conclusion

To the best of our knowledge, this is the first study to investigate the impact of musical expertise on line estimation abilities using both line section tasks and line extension tasks. Our findings indicate that musicians performed more accurately in bisecting and trisecting the lines compared to non-musicians. However, musicians’ overall line estimation abilities for extending lines were comparable to those of non-musicians, with the exception of better performance in the 1/2 extension condition. These results do not support the idea of a general, across-the-board enhancement of spatial cognition through music training. Instead, we propose that our findings may be explained by the combined effects of musicians’ superior spatial representation skills due to their musical expertise and the influence of the “enhancement” theory.

## Data availability statement

The raw data supporting the conclusions of this article will be made available by the authors, without undue reservation.

## Ethics statement

The studies involving humans were approved by East China Normal University Committee on Human Research Protection. The studies were conducted in accordance with the local legislation and institutional requirements. The participants provided their written informed consent to participate in this study.

## Author contributions

YP and ZW designed the study. YP and YH collected and analyzed the preliminary data for the study. YP, XiaL, and ZW wrote the manuscript. YP and ZX collected and analyzed the additional data. XinL, YB, and SK analyzed the additional data.
